# Optimal control and multitrophic physiologically‐based models: The binomial for successful decision support systems in insect pest management

**DOI:** 10.1002/ps.70974

**Published:** 2026-05-31

**Authors:** Luca Rossini, Ouassim Benhamouche, Emanuele Garone

**Affiliations:** ^1^ School of Agriculture, Policy and Development University of Reading Reading UK; ^2^ Service d'Automatique et d'Analyse des Systèmes Université Libre de Bruxelles Brussels Belgium

**Keywords:** model‐driven pest control, non‐linear programming problems, precision farming, physiologically‐based models, sustainable agriculture

## Abstract

**BACKGROUND:**

Agroecosystems can be viewed as dynamic systems that receive inputs through agronomic practices, generate yield as an output, and whose economic profitability depends on balancing production costs and revenues. Pests and pathogens reduce crop yield and often require growers to apply additional inputs, thereby increasing production costs. According to integrated pest management (IPM), however, maximising yield while minimising costs does not mean eradicating bio‐aggressors from the field but corresponds to maintain pest populations below economic thresholds. This objective can be achieved by identifying the optimal timing for control actions through decision support systems (DSSs) as, from a mathematical perspective, IPM can be formulated as an optimal control problem: the objective function accounts for economic returns and production costs, including treatments, and is driven by plant–pest dynamics.

**RESULTS:**

We introduced the essential concepts of an optimal control problem that faithfully fits the IPM guidelines. Multitrophic models account the interaction between plant and pest, while the effect of control actions relates to the objective function of the problem: the total economic income of the farm depends on the number of treatments and the timing of their application. Simulations showed both that lower pest population does not correspond to the maximum profit and that a good timing in the application can have almost the same effect of repeated treatments.

**CONCLUSION:**

This study revises the concept of DSS through optimal control applied to pest populations, pointing out that multitrophic models are fundamental to fit the IPM guidelines of economic and environmental sustainability. © 2026 The Author(s). *Pest Management Science* published by John Wiley & Sons Ltd on behalf of Society of Chemical Industry.

## Graphical abstract

Decision‐making in integrated pest management (IPM) corresponds to an optimisation problem where the plant–pest dynamics and the control actions are constraints and where the objective function accounts for the costs of the treatments and the total income at the harvest time. Notably, the lowest pest population within the field does not correspond to the highest profit, and the timing of the action is fundamental to maximise the efficacy of the control action.

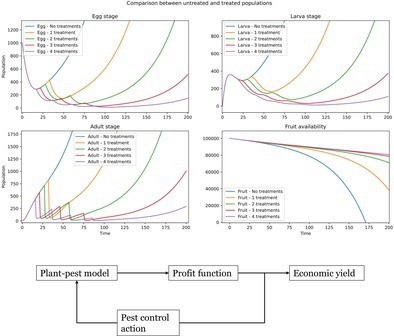

## 1 INTRODUCTION

The reduction of inputs and the optimisation of resources in cultivated fields are strongly driven by the synergy between new technologies and predictive capabilities.^1^ In this context, decision support systems (DSSs) represent one of the core innovations underpinning precision agriculture, as they can forecast future scenarios in cultivated fields to drive control actions.^2^ The predictive capability of DSSs relies on mathematical models that synthetically describe the main biological processes involved, together with the interactions among species and their relationships with the external environment.^3^, ^4^

The growing demand for DSSs has encouraged researchers worldwide to develop increasingly accurate models with stronger biological relevance and closer links to variables that can be directly measured in the field.^5^ In parallel, recent advances in sensing technologies and metrology applied to agriculture and biological systems have improved the reliability of the model predictions, as field data can now be actively integrated into models to update and refine simulations.^6^, ^7^, ^8^, ^9^ This aspect is particularly important, since the estimation of initial conditions and the continuous alignment of simulations with real field conditions remain among the main limitations to the widespread adoption of DSSs.^7^, ^8^, ^9^

DSSs can support a wide range of agronomic practices, including irrigation, fertilisation, and plant protection against insect pests and plant diseases.^10^, ^11^, ^12^ Phytosanitary emergencies are among the main drivers of both environmental pollution, particularly when managed through conventional agrochemicals, and increases in production costs. For this reason, considerable research has focused on the development of mathematical models describing insect population dynamics. These models form the core for DSSs, as they describe the evolution of pest outbreaks in cultivated fields and glasshouses.

Ideally, a DSS should reproduce *in silico* the integrated pest management (IPM) process, which promotes the use of control methods only where and when they are needed, following a ‘lowest impact philosophy’. Accordingly, DSSs should identify the periods of the growing season with the highest risk of outbreaks and simulate the effects of different control strategies on pest populations, ranging from methods with the lowest to the highest impact on environmental, animal, and human health. From an entomological perspective, treatments are applied when a specific threshold, defined for each crop–pest system, is exceeded. These thresholds are generally designed to ensure that the cost of each treatment remains economically justified with respect to the potential yield loss caused by pest activity.^13^ However, the identification of the optimal timing for interventions still largely relies on empirical thresholds based on monitoring data, such as the number of insects captured in traps, rather than on quantitative and economically driven information.

The difficulty of defining the optimal balance between production costs and yield returns has often led to a misunderstanding of pest control, shifting the focus from ‘a population level low enough to ensure the maximum economic income’ to ‘minimising the pest population into the fields’. This misconception has strongly affected the development of DSSs over the years, as the idea that these tools can be built solely on pest population dynamics models remains widespread among both researchers and practitioners. However, this approach creates a divergence between DSSs and the principles of IPM, as decisions are then based exclusively on pest abundance rather than on economic criteria. Actually, single‐species models are not sufficient to support an ideal model‐driven IPM framework through DSSs, since the interaction between the resources (the host plant/crop), the pest population, and the control action (whether conventional or based on natural enemies) is fundamental for addressing the problem using an economic perspective.

This work aims to show that a DSS should be formulated as an optimisation problem, as already highlighted in the literature,^5^, ^13^, ^14^, ^15^ in which the pest model represents only one component of the overall mathematical framework. Notably, the proposed approach accounts for plant–pest interactions and for the effects of control actions on both insect population dynamics and economic returns at harvest. The cost–benefit balance associated with control actions is included into the objective function of the problem, introducing a perspective that has so far been only partially explored in the existing literature.

As a result, the optimisation problem identifies the optimal timing for treatment applications in accordance with the principles of IPM and precision agriculture. Accordingly, the output of a DSS can be interpreted as a what if scenario driven by the main interacting actors and environmental factors. To illustrate the conceptual formulation of the optimisation problem that can be implemented within DSSs for pest management, a simple case study is presented, without loss of generality. Hence, two key aspects will be highlighted: (i) the main components of the framework, and (ii) the practical implementation of the problem in a real‐world scenario.

## 2 MATERIALS AND METHODS

Let us consider, without loss of generality, the simple case of a crop–pest system developing under constant environmental conditions of temperature and relative humidity, such as a crop cultivated in a glasshouse environment. In what follows, we consider a host plant whose fruits represent the substrate for pest oviposition. From a modelling perspective, this interaction can be described as a predator–prey system, where the plant acts as the prey and the pest as the predator.^16^, ^17^, ^18^

### 2.1 Plant–pest model

As a realistic case, we consider an insect pest with parthenogenetic reproduction (only females) whose life cycle is composed of three stages, (i.e., egg $E\left(t\right)$, preimaginal stages $L\left(t\right)$, and adult $A\left(t\right)$). The pest lays the eggs on the fruits of the host plant; accordingly, we can assume that for each egg laid, the overall field production is reduced of one fruit. This case is typical of many pests, such as *Drosophila suzukii* ^19^, ^20^ or *Bactrocera oleae*,^21^, ^22^ to give some relevant examples; moreover, it is worth pointing out that neither the stage division nor the sex ratio is affecting the *Principia* behind the present study.

For the sake of simplicity, we assume that the total number of fruits produced can be estimated by dividing the total weight of the fruits in a field (e.g., tons per hectare) by the average weight of a single fruit. To model the fruit dynamics two main aspects are considered:

1. Even in case of no pests within the field, plants drop a percentage of their fruits, depending on physiological aspects or other sources of stress.^23^, ^24^, ^25^ From a mathematical point of view, the fruit drop can be translated in a natural mortality rate $M_{\mathrm{fn}}$ that is affecting the population of fruits over time, $F\left(t\right)$.
2. As in a predator–prey system, additional loss of fruits is caused by oviposition. We mathematically describe oviposition through a second mortality rate β, which corresponds to the oviposition rate (reproduction rate) of the insect pest and that is proportional to the number of adult insects $A\left(t\right)$.

The stage‐population dynamics of the pest, instead, can be described with a compartmental model, as in the literature,^5^, ^26^, ^27^, ^28^, ^29^, ^30^, ^31^ where the transitions between the life stages i are ruled by development, $G_{i}$, and mortality, $M_{i}$, rates, respectively.

The previous discussion leads to the following difference equation model:

$$E\left(t+1\right)=\beta \;A\left(t\right)+\left(1-G_{E}\right)\;E\left(t\right)-M_{E}\;E\left(t\right)$$

$$L\left(t+1\right)=G_{E}\;E\left(t\right)+\left(1-G_{L}\right)\;L\left(t\right)-M_{L}\;L\left(t\right)$$

$$A\left(t+1\right)=G_{L}\;L\left(t\right)+\left(1-M_{A}\right)\;A\left(t\right)$$

(1) $$F\left(t+1\right)=\left(1-M_{\mathrm{fn}}\right)\;F\left(t\right)-\beta \;A\left(t\right)$$

For the sake of this work, we consider a discrete‐time model, as switching to continuous‐time and more complex models does not cause loss of generality in the methodology hereby presented.

### 2.2 Pest control action model

Equation (1) can be written in matrix form as

(2) $$x\left(t+1\right)=A\;x\left(t\right)+B\;u\left(t\right)$$

where $x\left(t\right)={\left(E\left(t\right),L\left(t\right),A\left(t\right),F\left(t\right)\right)}^{T}$ is the state vector of the populations (the superscript symbol *T* indicates the transpose operation) and

(3) $$A=\left(\begin{array}{cccc} 1-G_{E}-M_{E} & 0 & \beta & 0\\ G_{E} & 1-G_{L}-M_{L} & 0 & 0\\ 0 & G_{L} & 1-M_{A} & 0\\ 0 & 0 & -\beta & 1-M_{\mathrm{fn}} \end{array}\right)$$

is the matrix of the coefficients. The last term of Eqn (2) describes the external inputs to the system, which, in the context of pest management, correspond to control actions. In this study, we consider a treatment (either based on conventional agrochemicals or biopesticides) that specifically targets the adult population. This assumption is represented by setting the vector $B={\left(0,0,1,0\right)}^{T}$. Within this framework, the function $u\left(t\right)$ describes the number of adults added or removed from the system at time t. A control action applied at time $T_{i}$ affects the pest population through a time‐dependent mortality that gradually decays over time. In this work, the effect of a treatment is modelled as a decaying mortality characterised by the parameters $M_{T}\in \left(0,1\right)$ and $M_{C}\in \left(0,1\right)$. Mathematically the input assumes the following form

(4) $$u\left(t\right)=-\eta \left(t,T_{i}\right)\left(M_{T}M_{C}^{t-T_{i}}\right)B^{T}x\left(t\right)$$

where the function η ensures that (i) the treatment is active only at the time of the applications, $T_{i}$, and (ii) it has effect only for a limited number of days, $T_{W}$, after $T_{i}$,

(5) $$\eta \left(t,T_{i}\right)=\left\{\begin{array}{c} 0\;\text{if}\;t\notin \left[T_{i},T_{i}+T_{W}\right]\\ 1\;\text{if}\;t\in \left[T_{i},T_{i}+T_{W}\right] \end{array}\right.$$

This part completes the description of the plant–pest system that will be considered to introduce the optimisation problem and its connection with DSS and IPM.

### 2.3 Optimisation problem and DSS

The core question in IPM concerns the identification of the optimal number of treatments N to be applied during a growing season, together with the best timing $T_{1},\ldots ,T_{N}$ for their application, so that the economic return of the farm is maximised.^32^ This objective is addressed through the optimisation problem described in this section, which combines the plant–pest model with the effects of control actions to maximise yield and profit while accounting for the costs associated with pest management strategies and the practical constraints of the farming process. Furthermore, we assume that both the harvest time $T_{H}$ and the total number of harvested fruits $F\left(T_{H}\right)$ are known. These quantities depend on the pest's predatory activity which can, in turn, be influenced by control actions. In real agricultural scenarios, pest management strategies are constrained by national and regional regulations,^33^, ^34^ as well as by technical and logistical limitations.^2^, ^32^ Accordingly, for a given crop, we may assume (i) a maximum number of treatments per season $N_{T}$; (ii) a minimum interval $\Delta T_{\min}$ between two consecutive treatments; and (iii) a minimum interval $\Delta T_{H}$ between the final treatment $T_{N}$ and the harvest time $T_{H}$.

The profit function J accounts for both the total cost of the treatments applied during the growing season and the economic return from fruit production at harvest. Notably, the treatment cost is expressed as $P_{i}\;N$, where $P_{i}$ represents the cost of a single treatment and N the number of treatments, while the economic return is represented by $R\;F\left(T_{H}\right)$, with R denoting the market value associated with the harvested fruits at time $T_{H}$. Based on the assumptions described earlier, the optimisation problem can therefore be formulated as the following non‐linear integer programming problem:

$$\max_{\begin{array}{c} N\\ T_{1},\ldots ,T_{N} \end{array}}\;R\;F\left(T_{H}\right)-P_{i}\;N$$

subject to

$$x\left(t+1\right)=A\;x\left(t\right)+B\left(\sum_{i=1}^{N}-\eta \left(t,T_{i}\right)\left(M_{T}M_{C}^{t-T_{i}}\right)B^{T}\right)x\left(t\right)$$

$$N\leq N_{\max}$$

$$T_{i+1}-T_{i}\geq {\Delta T}_{\min},i=1,\ldots ,N-1$$

(6) $$T_{N}\leq T_{H}-{\Delta T}_{H}$$

### 2.4 Numerical solutions and comparison with other common pest control policies

The optimisation problem in Eqn (6) was solved using an *ad hoc* Python (version 3.13) script (publicly available at https://github.com/lucaros1190/ThreeForLoops) based on the *numpy* (version 2.2), *scipy* (version 1.15.3), and *multiprocess* (version 3.13.3) packages. Briefly, the script explores all the possible combinations of treatment timings, excluding periods during which plants are already covered by a previous treatment. For the sake of this study, two growing seasons were considered, with harvest times of $T_{H}=120$ and $T_{H}=200$ days, respectively, and with a maximum of four treatments allowed. Each treatment was assumed to remain effective for 15 days, meaning that two consecutive applications had to respect at least the minimum interval. The model parameters adopted in this study are reported in Table 1.

**Table 1:** List of the parameters considered to explore the behaviour of the optimisation problem (Eqn (5)) considered in this study

Parameter	Value
Initial conditions	$E\left(0\right)=1000$
	$L\left(0\right)=0$
	$A\left(0\right)=10$
	$F\left(0\right)=1.0\cdot 10^{5}$
Mortalities	$M_{E}=0.01$
	$M_{L}=0.01$
	$M_{A}=0.01$
	$M_{\mathrm{fn}}=0.001$
Development	$G_{E}=0.1$
	$G_{L}=0.1$
Fertility/predation rate	β=0.07
Efficacy of the treatment	$M_{T}=0.9$
Decay of the treatment	$M_{C}=0.8$
Price of a single treatment	$P_{i}=50$
Price of a single fruit	R=0.01

*Note:* The parameters were the same for the two‐harvest time scenarios explored: $T_{H}=120$ and $T_{H}=200$ days.

To show the behaviour of the optimisation problem, solutions corresponding to no treatment, one, two, three, and four treatments were compared, with the associated profit function evaluated in each case. In addition, for every treatment scenario, the resulting profit was compared with that obtained under time‐homogeneous applications, namely treatments applied at regular intervals throughout the growing season. In this latter case, treatment timings were determined by dividing the duration of the growing season by the number of treatments, while ensuring compliance with the pesticide coverage period before harvest. These comparisons were carried out to demonstrate that the optimisation framework can provide advantages both in reducing the number of treatments and in identifying the optimal timing for their application.

To improve computational efficiency, the exploration of treatment combinations was parallelised. The code was also designed to accommodate more complex models and a generic number of treatments, including possible release of natural enemies.

## 3 RESULTS

The values of the profit function obtained under time‐homogeneous treatment applications, reported in Table 2, can be compared with the results of the optimisation problem (Eqn (6)) shown in Table 3 for the two growing seasons of 120 and 200 days, respectively. Although the two scenarios highlight the same general patterns, their effects differ in magnitude, as longer growing seasons expose fruits to pest activity for a greater period of time. The results for the 120‐day and 200‐day growing seasons are presented separately.

**Table 2:** Results of the time‐homogeneous treatments for the different number of control actions hypothesised

Simulation	Harvest time 120 days		Harvest time 200 days	
	Treatment application time	Profit function value	Treatment application time	Profit function value
No treatment	—	J=686.12	—	J=0
One treatment	$T_{1}=60$	J=767.62	$T_{1}=100$	J=263.82
Two treatments	$T_{1}=52$	J=738.78	$T_{1}=92$	J=361.02
	$T_{2}=104$		$T_{2}=184$	
Three treatments	$T_{1}=35$	J=715.64	$T_{1}=61$	J=546.97
	$T_{2}=70$		$T_{2}=122$	
	$T_{3}=105$		$T_{3}=183$	
Four treatments	$T_{1}=26$	J=675.99	$T_{1}=46$	J=566.06
	$T_{2}=52$		$T_{2}=92$	
	$T_{3}=78$		$T_{3}=138$	
	$T_{4}=104$		$T_{4}=184$	

*Note:* In this case, treatments are applied at regular intervals, dividing the duration of the growing season by the number of treatments and respecting the pesticide coverage before the harvest. The total profit is expressed in euro (or any other currency).

**Table 3:** Results of the optimisation problem for the different number of treatments hypothesised

Simulation	Harvest time 120 days			Harvest time 200 days		
	Best treatment timing	Profit function value	Economic advantage with respect to time‐homogeneous treatments	Best treatment timing	Profit function value	Economic advantage with respect to time‐homogeneous treatments
No treatment	—	J=686.12	—	—	J=0	*—*
One treatment	$T_{1}=26$	J=784.80	+2.1%	$T_{1}=32$	J=334.94	+21.2%
Two treatments	$T_{1}=19$	J=770.33	+4.1%	$T_{1}=27$	J=611.34	+40.9%
	$T_{2}=43$			$T_{2}=57$		
Three treatments	$T_{1}=13$	J=729.73	+1.9%	$T_{1}=21$	J=637.49	+14.1%
	$T_{2}=31$			$T_{2}=46$		
	$T_{3}=53$			$T_{3}=75$		
Four treatments	$T_{1}=10$	J=682.84	1.0%	$T_{1}=16$	J=606.96	+6.7%
	$T_{2}=25$			$T_{2}=36$		
	$T_{3}=41$			$T_{3}=59$		
	$T_{4}=62$			$T_{4}=86$		

*Note:* The table lists the best combination of treatment times, the corresponding profit at the end of the season, and the profit increase (in percentage) with respect to the case of time‐homogeneous control actions (Table 2). The total profit is expressed in euro (or any other currency).

### 3.1 Scenario 1: growing season of 120 days

This scenario provides initial evidence that the optimisation problem (Eqn (6)) yields economic benefits, as it shows that applying treatments at optimal times increases profit. The values reported in Tables 2 and 3 indicate an average profit increase of approximately 2% compared with time‐homogeneous treatments. This improvement is more pronounced for lower numbers of treatments, suggesting that as N increases, the two strategies tend to produce increasingly similar outcomes. In both cases, however, there is a point beyond which additional treatments no longer provide economic benefit. This result highlights that increasing the number of treatments no longer provide an economic benefit, and that a threshold exists beyond which further control actions are no longer economically justified.

**Figure 1:**
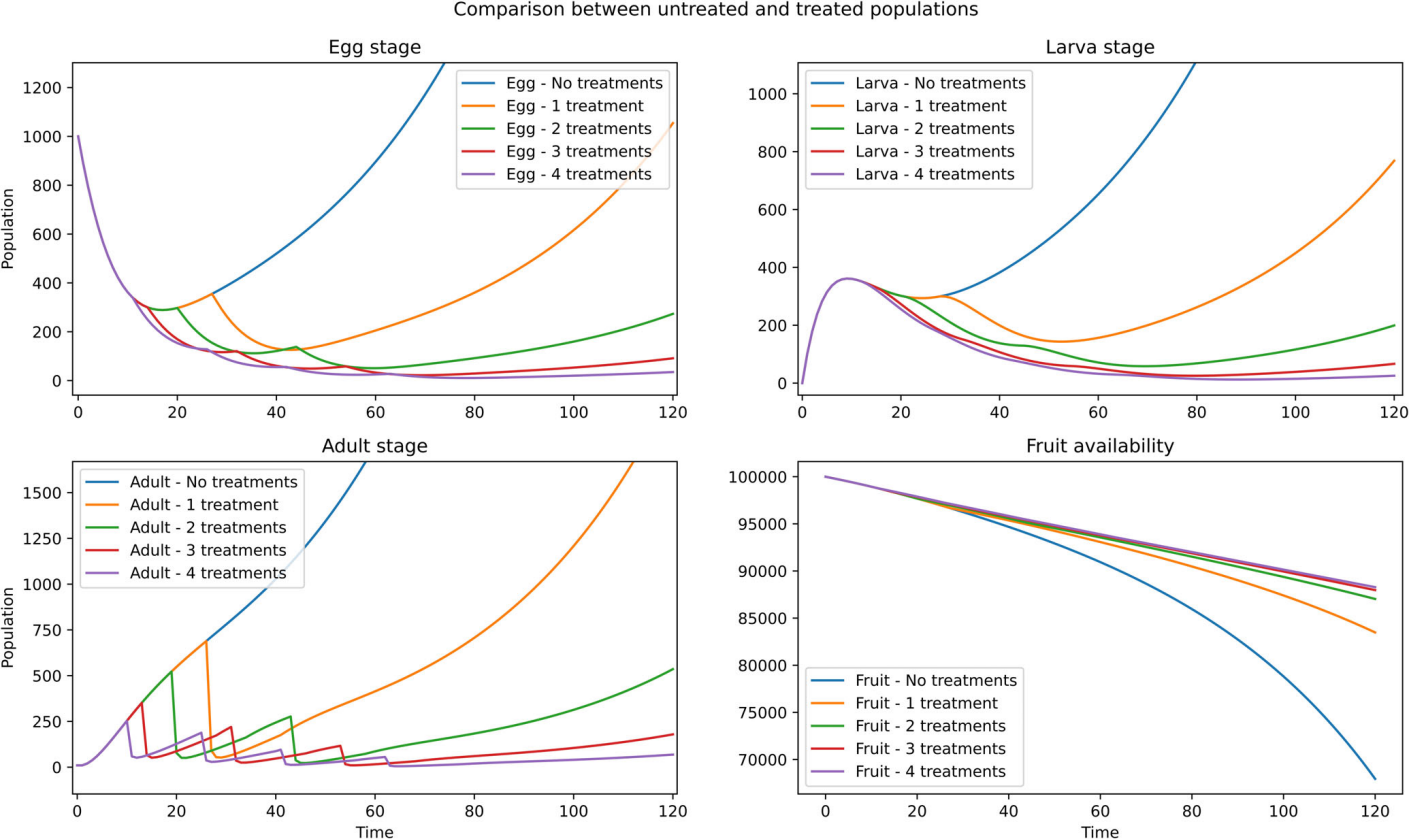
Population dynamics of eggs, larvae, adults, and fruits with no treatment, one, two, three, and four treatments applied to the best timings, according to the solutions of the problem (Eqn (5)). This is the case in which the growing season is 120 days long ($T_{H}=120$).

The optimisation problem (Eqn (6)) indicates that the lowest economic profit is obtained when four treatments are applied. The difference in profit between one and two treatments is minimal, suggesting that the second treatment does not generate sufficient additional benefit to justify its cost. In fact, a single treatment over the season represents the most economically efficient strategy, as additional treatments (two, three, or four) reduce pest populations to lower levels (Fig. 1) without delivering corresponding economic gains. This result is consistent with the time‐homogeneous case, however, optimal timing is crucial in achieving higher profitability. In general, treatments tend to be concentrated in the early part of the growing season, when pest populations are assumed to increase in relation to fruit availability. As the number of treatments increases, the timing of the first application is brought forward, in some cases occurring close to the sowing date.

### 3.2 Scenario 2: growing season of 200 days

This scenario differs from the previous one, as the lowest yield was obtained in the absence of treatments, and the profit increase achieved through optimisation was considerably greater than in the case of time‐homogeneous applications. The advantage provided by optimal timing reached a maximum increase of 40.1% in the case of two treatments. However, similarly to the 120‐day scenario, the economic benefit decreased as the number of treatments increased, both for time‐homogeneous applications and for the optimisation problem (Eqn (6)).

**Figure 2:**
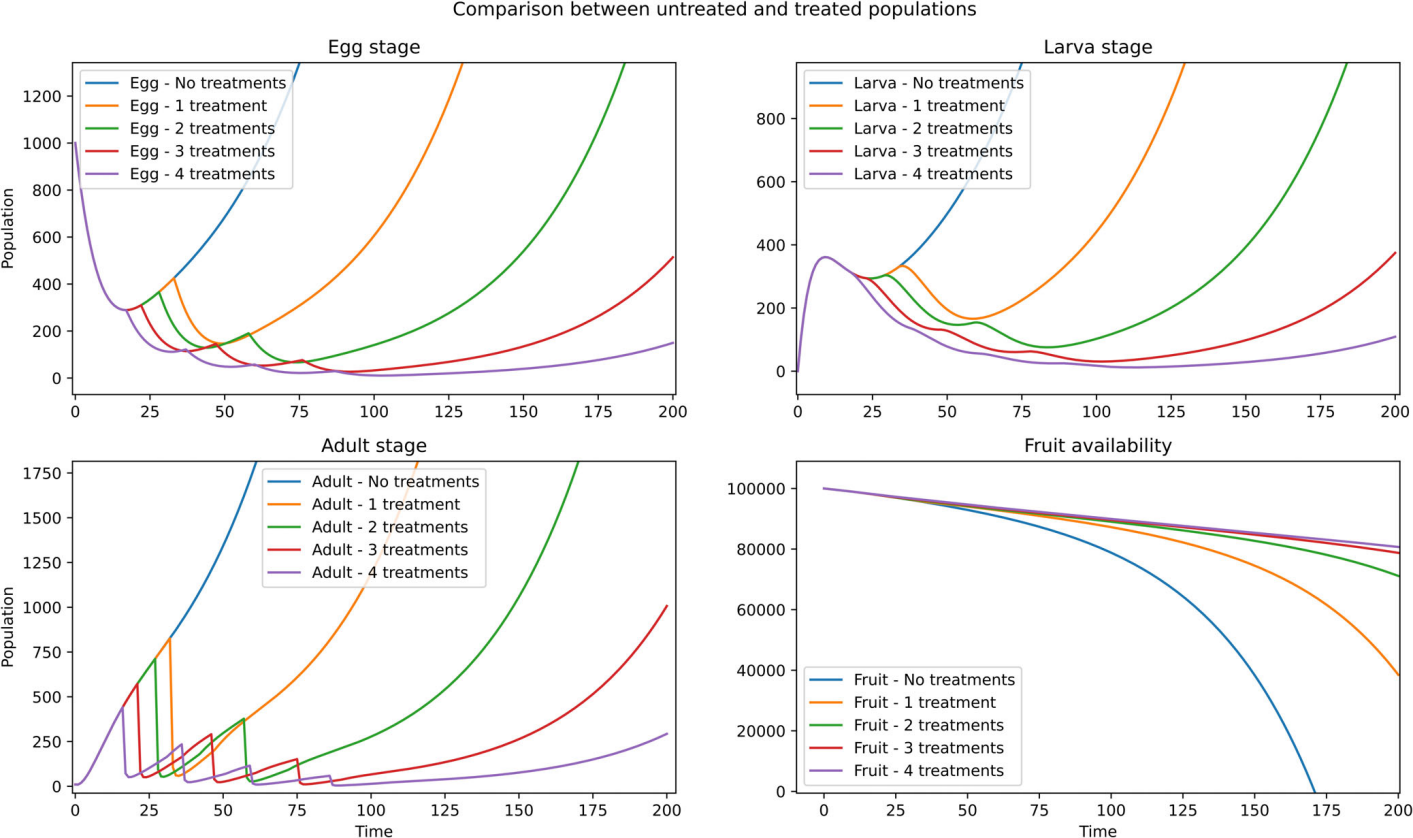
Population dynamics of eggs, larvae, adults, and fruits with no treatment, one, two, three, and four treatments applied to the best timings, according to the solutions of the problem (Eqn (5)). This is the case in which the growing season is 200 days long ($T_{H}=200$).

According to the values reported in Tables 2 and 3, the highest profit (Fig. 2) was achieved with three treatments applied at the optimal timings, while two optimally timed treatments generated a higher profit than four time‐homogeneous applications. As in the previous scenario, treatments were mainly concentrated in the early part of the growing season, with the first application occurring progressively closer to the sowing date as the number of treatments increased. Overall, this scenario further confirms and more clearly highlights the advantages of using the optimisation framework to guide pest management decisions.

## 4 DISCUSSION AND CONCLUSIONS

This study revised the concept of decision support systems by introducing an optimisation framework aimed at maximising the farm's economic return, in closer accordance with the principles of IPM. Although the concepts were presented through a simplified case study, the framework is sufficiently general to be extended to more complex scenarios involving advanced models and multiple trophic interactions. The simulations highlighted that a higher number of treatments may reduce pest populations within the field, but does not necessarily result in greater economic returns once production costs are taken into account. Furthermore, this work emphasises the importance of treatment timing, showing that higher profits can be achieved with fewer applications, when control actions are carried out at the most appropriate moments.

The example considered in this study confirms that the formulation of an optimisation problem can effectively reproduce the principles of IPM. In particular, the scenario described in this article is representative of a glasshouse cultivation system exposed to pest infestations. Under these conditions, environmental variables are assumed to remain approximately constant, and consequently the model coefficients can also be treated as constant. Within this framework, the optimal control problem (Eqn (6)) identifies the best timing for the application of control actions and, depending on the type of treatment considered, determines the number of applications that maximises the economic profit.

The main aspect we would like to emphasise is the introduction of an objective function based on the balance between production costs and economic returns, which represents the key novelty of the proposed framework. Optimal control and non‐linear programming approaches are not new in pest management, as these concepts have been introduced by Plant^35^ to support eradication programmes. Over the years, several authors proposed similar approaches accounting for either the combined effects of different control strategies, such as conventional pesticides and predators/parasitoids, or for more complex models describing pest stage dynamics. This body of literature has provided a solid foundation for our work, as incorporating the effects the control actions is essential for developing realistic and reliable DSS frameworks.

Modelling the effect of pest control actions represent a fundamental step towards the development of reliable DSSs. Biological control based on predators and parasitoids can be described through Volterra–Lotka‐type predator–prey models,^36^ as previously demonstrated in the literature.^37^, ^38^, ^39^ An example of the combined use of multiple control strategies was provided by Pei *et al*.,^40^ who investigated the interactions between natural enemies, pest populations, and conventional pesticides. This body of literature provides a valuable basis for improving the representation of pest dynamics in the field, to which the host plant dynamics should also be connected.

To the best of our knowledge, neither host plant dynamics nor the economic return associated with harvest have previously been included into optimal control problems applied to insect pests, despite representing key components of IPM. In earlier studies, objective functions generally accounted for treatment costs, pest‐induced damage, or economic thresholds. For example, Bliman *et al*.^41^ introduced an optimal control framework for mosquito management based on the release of sterile males, where the objective function aimed to reduce the wild male population while minimising both the duration and the overall costs of the control programme. Although this application lies outside the agronomic context, the conceptual framework proposed in Eqn (6) could complement the approach of Bliman *et al*.^41^ by including the economic costs associated with human and animal infections caused by mosquitoes.

An interesting application combining sterile male releases with insecticide‐based treatments was presented by Hackett and Bonsall.^13^ In this case, the objective function is more comprehensive, as it accounts for both treatment costs the economic damage. However, the host plant is not explicitly included either in the model or in the objective function, thus neglecting the dynamics of economic returns as an effect of pest activity and control measures. Future developments of the objective function in optimisation problem (Eqn (6)) could draw inspiration from the work of Hackett and Bonsall,^13^ as integrating the two approaches may lead to a more comprehensive framework capable of accounting for multiple treatments and multitrophic interactions.

Optimal control approaches may also prove valuable for managing primary outbreaks of alien species accidentally introduced into non‐native areas. This concept was previously proposed by Edholm^42^ through a general framework, although without including the host plant within the model. In such scenarios, optimisation problem (Eqn (6)) could still be applied, even though the control strategy would be primarily focused on minimising pest populations rather than maximising economic return.

The interaction between the crop and its pest provides a more realistic and dynamic representation of the cost–benefit balance that the optimal application of control strategies should maximise. More generally, the objective function can be extended to account for treatments targeting different life stages, while the model describing plant–pest dynamics can be further enriched by including additional trophic interactions, such as natural enemies (predators/parasitoids) acting as biological control agents. Within this perspective, our study may be viewed as a conceptual extension of the framework proposed by Rafikov and Balthazar,^43^ who introduced an optimal control problem for biological control based on predators. However, their objective function did not include the crop's economic return and neglected host plant dynamics, which represent essential components of a DSS consistent with IPM principles.

The quality of model predictions can be further improved by including estimators capable of correcting simulations on the basis of field measurements. This theoretical framework was previously introduced by Bono Rosselló *et al*.,^6^, ^7^ who demonstrated that the use of an extended Kalman filter can substantially reduce the dependency of the model on the initial conditions while improving prediction accuracy. This body of literature represents an additional component that future developments should consider to further enhance the reliability of the optimisation problem (Eqn (6)).

Optimal control applied to insect pest populations is becoming an increasingly valuable tool for the development of reliable DSS consistent with IPM guidelines. Through a simple yet realistic example, this study showed both the key elements that should be considered when formulating the optimisation problem and its capability to reproduce the underlying logic of IPM. We believe that this theoretical framework can be further developed and adapted to a wide range of applications, including plant pathology, thereby providing effective support for precision agriculture and pesticide reduction strategies.

## Data Availability

Data sharing not applicable to this article as no datasets were generated or analysed during the current study.
